# AURKB promotes bladder cancer progression by deregulating the p53 DNA damage response pathway via MAD2L2

**DOI:** 10.1186/s12967-024-05099-6

**Published:** 2024-03-21

**Authors:** Linzhi Li, Pengcheng Jiang, Weimin Hu, Fan Zou, Ming Li, Ting Rao, Yuan Ruan, Weimin Yu, Jinzhuo Ning, Fan Cheng

**Affiliations:** Department of Urology, Renmin Hospital of Wuhan University, Wuhan, 430060 China

**Keywords:** Bladder cancer, Aurora kinase B, Mitotic arrest-deficient 2-like protein 2, p53

## Abstract

**Background:**

Bladder cancer (BC) is the most common urinary tract malignancy. Aurora kinase B (AURKB), a component of the chromosomal passenger protein complex, affects chromosomal segregation during cell division. Mitotic arrest-deficient 2-like protein 2 (MAD2L2) interacts with various proteins and contributes to genomic integrity. Both AURKB and MAD2L2 are overexpressed in various human cancers and have synergistic oncogenic effects; therefore, they are regarded as emerging therapeutic targets for cancer. However, the relationship between these factors and the mechanisms underlying their oncogenic activity in BC remains largely unknown. The present study aimed to explore the interactions between AURKB and MAD2L2 and how they affect BC progression via the DNA damage response (DDR) pathway.

**Methods:**

Bioinformatics was used to analyze the expression, prognostic value, and pro-tumoral function of AURKB in patients with BC. CCK-8 assay, colony-forming assay, flow cytometry, SA-β-gal staining, wound healing assay, and transwell chamber experiments were performed to test the viability, cell cycle progression, senescence, and migration and invasion abilities of BC cells in vitro. A nude mouse xenograft assay was performed to test the tumorigenesis ability of BC cells in vivo. The expression and interaction of proteins and the occurrence of the senescence-associated secretory phenotype were detected using western blot analysis, co-immunoprecipitation assay, and RT-qPCR.

**Results:**

AURKB was highly expressed and associated with prognosis in patients with BC. AURKB expression was positively correlated with MAD2L2 expression. We confirmed that AURKB interacts with, and modulates the expression of, MAD2L2 in BC cells. AURKB knockdown suppressed the proliferation, migration, and invasion abilities of, and cell cycle progression in, BC cells, inducing senescence in these cells. The effects of AURKB knockdown were rescued by MAD2L2 overexpression in vitro and in vivo. The effects of MAD2L2 knockdown were similar to those of AURKB knockdown. Furthermore, p53 ablation rescued the MAD2L2 knockdown-induced suppression of BC cell proliferation and cell cycle arrest and senescence in BC cells.

**Conclusions:**

AURKB activates MAD2L2 expression to downregulate the p53 DDR pathway, thereby promoting BC progression. Thus, AURKB may serve as a potential molecular marker and a novel anticancer therapeutic target for BC.

**Supplementary Information:**

The online version contains supplementary material available at 10.1186/s12967-024-05099-6.

## Background

Bladder cancer (BC) is the most common malignancy of the urinary system and the fourth most common malignancy in men. It includes non-muscle-invasive bladder cancer (NMIBC), with a high recurrence rate, and muscle-invasive bladder cancer (MIBC), with high invasiveness and mortality rates [1]. Although intravesical Bacillus Calmette-Guerin (BCG) remains the mainstay of therapy for NMIBC, many intermediate- and high-risk NMIBC patients are either refractory to it or may experience disease relapse. The therapeutic options for MIBC have expanded to include immunotherapy with checkpoint inhibitors, targeted therapies, and antibody–drug conjugates [2–4]. Therefore, novel therapeutic targets and treatment options need to be identified.

Aurora kinase B (AURKB) is a mitotic serine/threonine protein kinase belonging to the aurora kinase family. As a member of the chromosomal passenger protein complex, AURKB is involved in cell cycle progression [5]. AURKB is overexpressed in a wide range of malignancies, including colorectal cancer [6], prostate cancer [7, 8], human seminoma [9], and thyroid carcinoma [10]. Accumulating evidence indicates that AURKB is associated with tumor development, invasion, metastasis, poor prognosis, and drug resistance in various malignancies [11]. Targeting AURKB is increasingly being considered a feasible therapeutic strategy against various tumors [12]. The currently available AURKB inhibitors have shown therapeutic efficacy both in vitro and in vivo [11]. Recent studies have revealed that AURKB is regulated by activators, such as Myc and cyclin K, and that it also regulates certain proteins, such as c-Myc and p53 [13–15]. However, the biological functions of AURKB in BC and the molecular mechanisms underlying these functions have not yet been fully investigated.

Mitotic arrest deficient 2-like protein 2 (MAD2L2), also known as REV7, is identified as a subunit of DNA polymerase ζ [16] and a component of the mitotic spindle assembly checkpoint [17]. As a versatile protein, MAD2L2 interacts with various proteins and forms different protein complexes, thereby playing vital roles in DNA damage repair, cell cycle regulation, and carcinogenesis [18]. Similar to AURKB, MAD2L2 is dysregulated in different cancer cell lines and tumor tissues; MAD2L2 overexpression is associated with poor prognosis in various cancer types [18]. Several studies have reported the coactivity of AURKB and MAD2L2 in cancer cells [19]. Moreover, both AURKB and MAD2L2 are involved in mitosis, cell cycle checkpoints, and DNA damage response (DDR). However, there is a lack of mechanistic studies revealing the potential interactions and biofunctions between AURKB and MAD2L2 in cancer biology in BC cells.

The tumor suppressor protein p53 plays a significant role in maintaining genomic stability and the DDR. In the p53 DDR pathway, DNA replication stress, which results in DNA double-strand breaks (DSBs) activates p53 [20, 21]. Activated p53 upregulates the transcription of its downstream targets, causing cell cycle arrest, apoptosis, and senescence [22]. Studies have shown that AURKB suppresses the activity of p53 via the phosphorylation of Ser183, Thr211, and Ser215, which quickens its degradation by the proteasome [23]. Moreover, recent studies have revealed that MAD2L2 blocks homologous recombination and promotes nonhomologous end joining by inhibiting the 5' end resection downstream of 53BP1 and RIF1 and plays a role in determining the choice of DSBs repair pathway to be followed [24]. Taken together, these findings indicate a significant connection between AURKB, MAD2L2, and the p53 DDR pathway in BC cells; this aspect requires further investigations.

In the present study, we explored the mechanisms underlying the oncogenic activity of AURKB in BC. We investigated the mechanisms whereby AURKB regulates MAD2L2 expression and promotes BC progression via the p53 DDR pathway.

## Materials and methods

### Data mining

The gene expression RNA sequencing data and corresponding clinical data were downloaded from The Cancer Genome Atlas (TCGA) database (https://portal.gdc.cancer.gov/). Datasets of the GSE13507 cohort were downloaded from the Gene Expression Omnibus (GEO) database (https://www.ncbi.nlm.nih.gov/geo). Tissue samples and clinical data of 10 BC patients were obtained from Renmin Hospital of Wuhan University. The Ethics Committee of Medical School of Wuhan University approved this study. All patients participating in the study obtained informed consent.

### Bioinformatics analyses

The level of AURKB in pan-cancer was analyzed by tumor immune estimation resource (TIMER) 2.0 database (http://timer.cistrome.org/). The FPKM data from TCGA and GEO databases were transformed using log2 (TPM + 1) for further calculation. We used R 4.2.1 for further data processing and statistical analysis. To verify the prognostic value of AURKB and MAD2L2, we used the survival package to conduct Kaplan–Meier univariate survival analysis based on the data in GEO and TCGA databases. The protein–protein interaction (PPI) network was constructed using STRING (https://string-db.org/) for searching a database of interacting genes. GO/KEGG term enrichment analyses were conducted using R packages. Gene set enrichment analysis (GSEA) was performed using the C2.cp.kegg.v2022.1.Hs.symbols.gmt with GSEA 4.3.0 software. In this study, we considered gene sets with nominal p-value < 0.05 and a false discovery rate (FDR) < 0.25 to be statistically significant.

### Cell culture

The human BC cell lines (T24 and 5637) were acquired from the Chinese Academy of Sciences (Shanghai, China). T24 and 5637 were cultured in RPMI-1640 medium (Pricella, Wuhan, China). All culture media contained 10% fetal bovine serum (Pricella) and 1% penicillin/streptomycin (Pricella). Cells were maintained at 37 °C in a humidified 5% CO2 incubator.

### Lentiviral transfection

For stable knockdown or overexpression of AURKB, MAD2L2 and p53, lentiviral-based shRNAs were synthesized by OBiO Co., Ltd (Shanghai, China). After transfection for 2–4 days, cells were selected in 4 µg/ml puromycin. Transfection was conducted following established protocols (Additional file 8: Methods section). The knockdown or overexpression efficiency was validated by Real-Time Quantitative PCR and western blot analysis.

### Quantitative reverse transcription polymerase chain reaction (RT-qPCR)

The total RNA from the patient samples and cells was extracted using TRIzol reagent (Servicebio, Wuhan, China) and then measured by NanoDrop 2000 (Thermo Fisher, USA). cDNA was generated using the SweScript All-in-One First-Strand cDNA Synthesis SuperMix for qPCR (Servicebio). RT-qPCR was performed with 2 × SYBR Green qPCR Master Mix (Servicebio) using the Lightcycler 4800II (Roche, Basel, Switzerland) following the manufacturer’s instructions. The relative gene expression was calculated using the 2 − ΔΔCt method and normalized to the expression of GAPDH. The specific RT-qPCR primer sequences were listed in Additional file 7: Table S1.

### Western blot analysis, immunofluorescence (IF), and immunohistochemistry (IHC) staining

For western blot analysis, cells were washed twice with PBS and lysed on ice in RIPA buffer (Servicebio) containing 0.1 mM PMSF (Servicebio) and phosphatase inhibitors (Servicebio). Equal amounts of protein samples were fractionated by SDS/PAGE and transferred to the PVDF membrane. After blocking with a protein-free fast blocking buffer (Servicebio), the membranes were incubated with primary antibodies overnight at 4 °C, followed by incubation with HRP-conjugated secondary antibodies (Servicebio) at room temperature for 1 h. All bands were measured using an ECL kit (Servicebio) by chemiluminescence (Bio-Rad) and analyzed using ImageJ software. The following primary antibodies were used in the present study: AURKB (1:1000, A1020, ABclonal, Wuhan, China), MAD2L2 (1:1000, A4630, ABclonal), Cyclin D1 (1:1000, A19038, ABclonal), p53 (1:1000, ab32049, Abcam, UK), p21 (1:1000, A1483, ABclonal), γH2A.X (1:5000, T56572, Abmart, Shanghai, China), β-Tubulin (1:5000, M20005, Abmart).

For IF staining, cells were fixed with 4% paraformaldehyde for 15 min at room temperature, permeabilized with 0.5% TritonX-100 for 15 min, blocked with 5% BSA for 1 h, and incubated with primary antibodies overnight at 4°C in a humidified box. After sufficient washing, cells were further incubated with fluorescence-conjugated secondary antibodies at room temperature for 1 h in the dark. Finally, nuclei were stained with DAPI. Fluorescence images were acquired using a fluorescence microscope (BX53, Olympus, Japan). Antibodies used in IF were as follows: KI67 (1:200, 27309-1-AP, Proteintech, Wuhan, China), AURKB (1:50, A19539, ABclonal), MAD2L2 (1:50, A4630, ABclonal).

For IHC staining, paraffin-embedded tissues were sliced into 5 µm thickness. The tissue sections were dewaxed and the endogenous peroxidase was blocked by 0.3% H_2_O_2_. After blocking with 5% BSA, the sections were incubated with primary antibody overnight at 4°C and incubated with horseradish peroxidase-conjugated secondary antibody for 1 h. The sections were subsequently reacted with DAB (Vector Laboratories) and counterstained with hematoxylin. The histochemistry score (H-score) of protein expression was calculated by multiplying the staining intensity (0, negative; 1, weak; 2, moderate; and 3, strong) with the percentage of positive cells (0, 0%-10%; 1, 11%-25%; 2, 26%-50%; 3, 51%-75%; and 4, > 75%). Antibodies used in IHC were as follows: AURKB (1:50, A19539, ABclonal), MAD2L2 (1:50, A4630, ABclonal), KI67 (1:50, 27309-1-AP, Proteintech), CyclinD1 (1:100, A11022, ABclonal), p53 (1:50, ab32049, Abcam).

### Co-immunoprecipitation (Co-IP) assay

Cells were lysed on ice in RIPA buffer containing 1% Cocktail (Servicebio). 10% input was saved, and the cell lysate was incubated with IgG (Proteintech) or primary antibody (AURKB, MAD2L2) at 4 °C overnight on a rotator, followed by Protein A + G beads (Beyotime, Shanghai, China) for another 2h of incubation. The supernatant was taken from the beads after centrifugation. The beads were washed three times using the inhibitor-containing lysate, and then boiled with Sample Loading Buffer (Beyotime) at 95 °C for 10 min before SDS-PAGE and western blot.

### CCK-8 assay

Cell viability was analyzed using the Cell Counting Kit-8 (CCK-8; Servicebio) in accordance with the CCK-8 assay protocols. Cells were seeded into 96-well plates at a density of 2 × 10^3^ cells/well. When cultured to 0, 24, 48, 72, and 96 h, CCK-8 (10 µL/well) was added to the cells and incubated at 37 °C for 1 h. We measured the absorbance of each well at 450 nm using a microplate reader (Bio-Rad Laboratories, Inc.).

### Cell cycle analysis and apoptosis assay

Briefly, cell cycle analysis was assessed with the propidium iodide (YEASEN) by flow cytometry (CytoFLEX, Beckman Coulter, USA) according to standard protocols. For cell apoptosis assay, cells were digested using Trypsin without EDTA. After being washed twice with PBS, cell apoptosis was measured by flow cytometry using Annexin V-FITC/PI Apoptosis Kit (Elabscience, Wuhan, China) according to manufacturer’s instructions. Data were analyzed using the FlowJo 10.6.2 software.

### Colony-forming assay

Cells were seeded into 6-well plates at a density of 2000 cells/well for a 14-day incubation period. After fixation in 4% paraformaldehyde and staining with crystal violet, pictures were taken and the colony formation rate was determined.

### Senescence-associated β-galactosidase (SA-β-gal) staining

Cells were fixed, and then incubated with SA-β-Gal staining solution (Beyotime) according to manufacturer’s instructions. Representative pictures were taken under bright-field microscopy (IX71, Olympus) and SA-β-gal^+^ cells rate was determined.

### Wound healing assay

Cells were seeded into 6-well plates and were cultured to confluency. Wounds were generated by scratching cell layer with 200 µL plastic pipette tips. The final images were taken and the gap distances of migrating cells were measured using a microscope (IX71, Olympus).

### Transwell cell invasion assay

Cell invasion assay was performed with Matrigel-coated transwell chamber (Corning, USA) according to the standard method. 600 µL of complete media was added to the lower chamber, and 200 µL of medium containing 1 × 10^4^ cells in serum-free RPMI-1640 was put in the upper chamber (8-mm pore size, Corning). The upper chamber was coated with 80 µl of the mixed solution of Matrigel and serum-free medium at a ratio of 1:8 (BD Biosciences). After being cultured for 48 h, cells were fixed in 4% paraformaldehyde and stained with crystal violet. Five fields per well were randomly selected and images were acquired by a microscope (IX71, Olympus).

### Nude mouse xenograft assay

Four-week-old Balb/c nude mice were purchased from the Centre of Experimental Animals at Wuhan University Medicine College (Hubei, China). All nude mice were kept in standard, infection-free housing conditions, and allowed to drink and eat freely. Each nude mouse received a subcutaneous injection of T24 cells (5 × 10^6^) resuspended in 100 µL of PBS. The growth of tumor size (L, longest dimension; W, shortest dimension) was evaluated by the vernier caliper every five days, and tumor volumes were calculated using the formula V = L x W x W/2. After 45 days, mice were euthanized and the tumors were then collected for additional research.

### Statistical analysis

All statistical analyses and data visualization were performed using GraphPad Prism 8 software. All data were presented as the mean ± SD at least three independent experiments. The Shapiro‒Wilk test was employed to assess normality. For the data with normal distribution, Student's t-test was used for comparisons between two independent groups. And one-way analysis of variance (ANOVA) with Bonferroni’s test was used to compare multiple groups of data. For the data with non-normal distribution, the Mann–Whitney U test was performed to analyze differences between two groups, and the Kruskal–Wallis H test, followed by Dunn's test was used for comparisons among multiple groups. Spearman’s correlation analysis was used to evaluate the relationship between the expressions of target genes. A p-value < 0.05 was considered statistically significant.

Detailed experimental procedures are described in the Additional file 8: Methods section.

## Results

### AURKB is highly expressed and associated with prognosis in BC

**Fig. 1:**
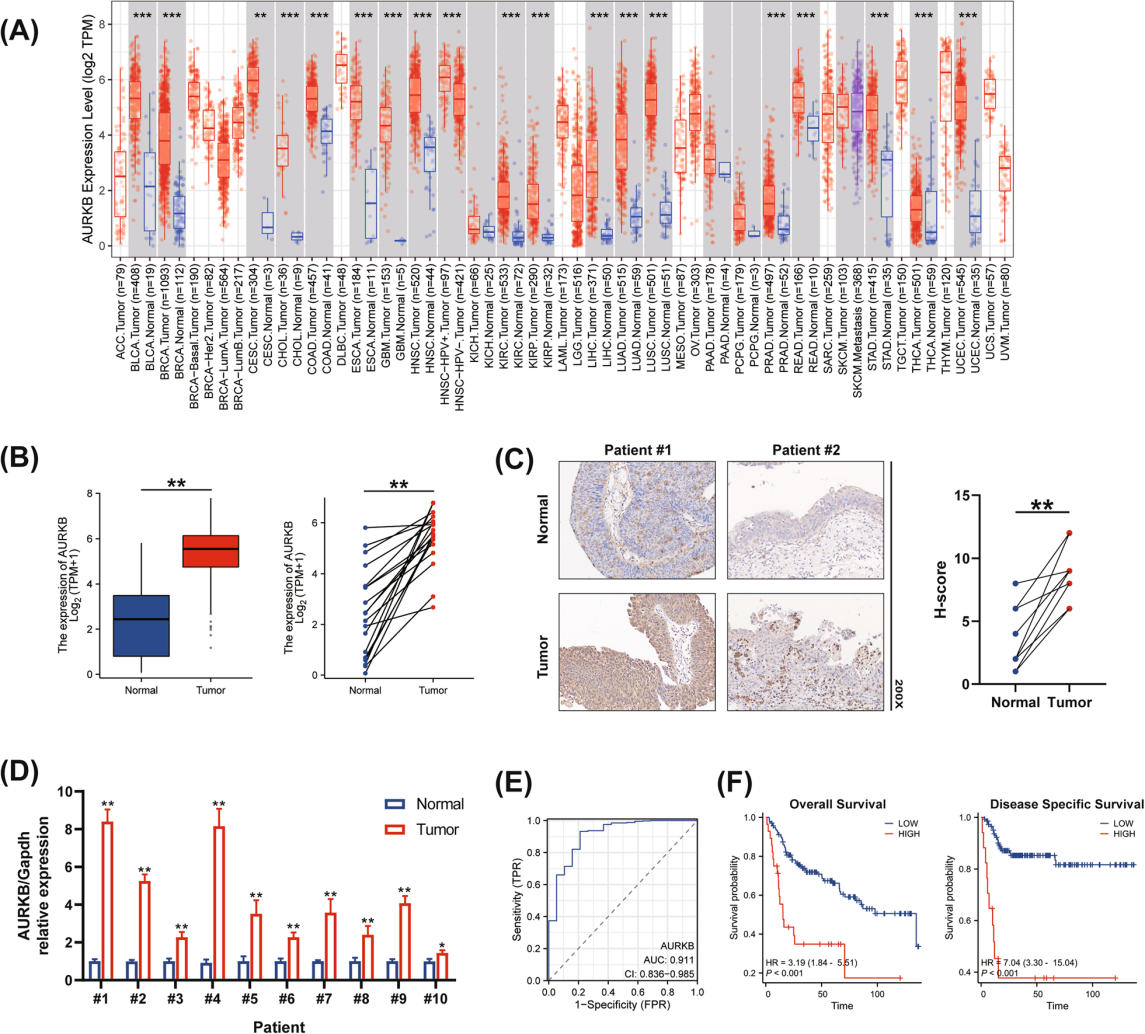
AURKB is highly expressed and is strongly associated with prognosis in BC. **A** Differential AURKB expression in various types of tumor tissues and adjacent normal tissues was analyzed by TIMER 2.0 database. **B** Differential AURKB expression in BC tissues and normal bladder tissues in unpaired and paired samples. **C** Representative IHC images of AURKB in BC tissues and adjacent normal tissues. H-score of AURKB expression in the clinical tumor and adjacent normal tissues of 10 BC patients. **D** RT-qPCR analyses of mRNA expression levels of AURKB between paired BC tissues and adjacent normal tissues. **E** The ROC curve analysis indicated the high-expression specificity of AURKB in tumor and normal tissues in TCGA. **F** Kaplan–Meier survival curves for OS and DSS comparing the high and low expression of AURKB in the GSE13507 database. (The magnification under the microscope is shown as marked in the figure. *p < 0.05, **p < 0.01, *** p < 0.001)

The TIMER 2.0 database was used to assess the expression level of AURKB in pan-cancer. The results showed that AURKB was highly expressed in pan-cancer (Fig. 1A). Meanwhile, RNA-seq data from TCGA was analyzed to demonstrate that AURKB is highly expressed in BC tissues compared with normal tissues in unpaired and paired samples (Fig. 1B). Furthermore, we performed immunohistochemistry staining for AURKB in 10 pairs of BC tissues and adjacent normal tissues (Fig. 1C). In addition, RT-qPCR was performed to detect the mRNA levels of AURKB in samples (Fig. 1D). As expected, the results revealed that AURKB expression was significantly up-regulated in BC tissues compared to normal tissues adjacent to the cancer, at both mRNA and protein levels.

Next, we explored the diagnostic and prognostic values of AURKB in BC. The results of ROC curves (Fig. 1E) revealed that the AUC reached 0.911(0.836–0.985, 95%CI, p < 0.01), which indicated that AURKB plays a significant role in BC diagnosis. For the prognostic potential of AURKB in BC, GSE13507 cohort databases were selected for this study. The Kaplan–Meier curve (Fig. 1F) showed that BC patients with high AURKB protein level had a worse overall survival (OS) and disease specific survival (DSS). Taken together, these findings suggest that AURKB, as an oncogene, plays a critical role in BC development.

### AURKB knockdown suppresses cell proliferation, cell cycle progression, migration and invasion, and upregulates cellular senescence

**Fig. 2:**
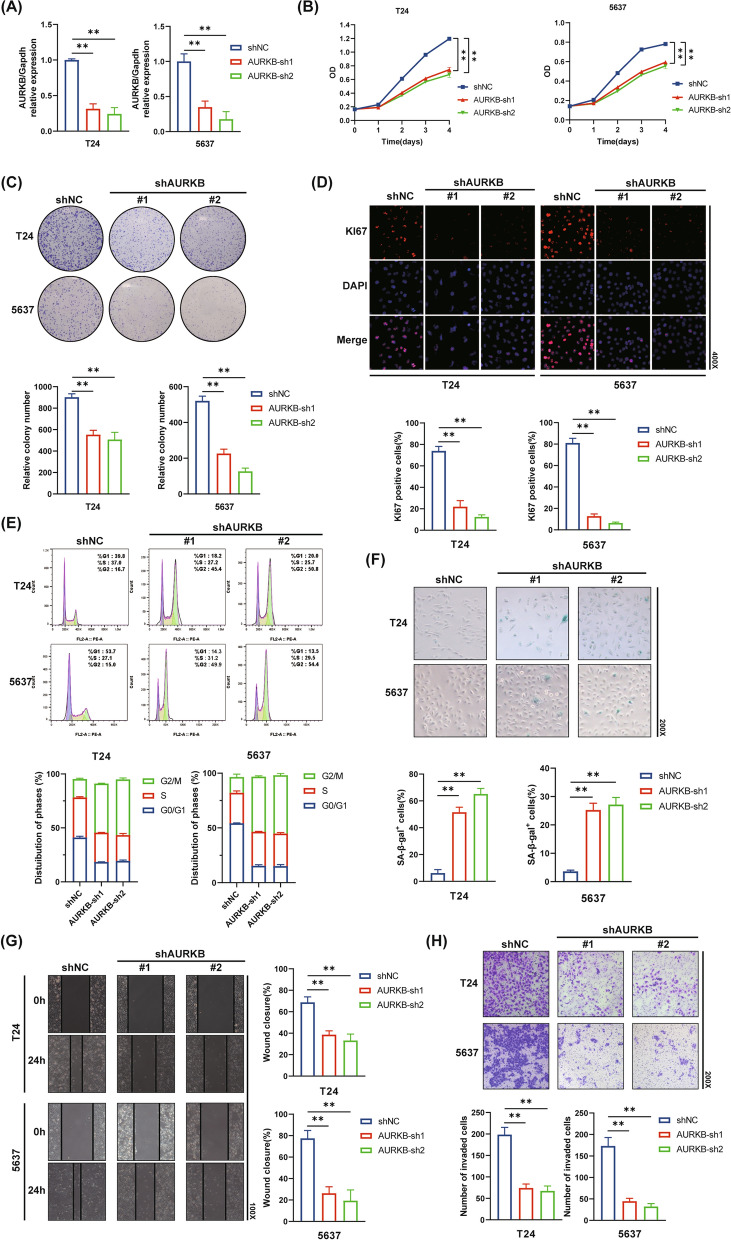
AURKB knockdown suppresses cell proliferation, cell cycle progression, migration and invasion, and upregulates cellular senescence. **A** The RT-qPCR analyses detected shRNAs transfection efficiency in T24 and 5637 cells. **B** Cell viability was measured by the CCK-8 assay in T24 and 5637 cells. **C** The colony-forming assay detected the proliferation ability of a single tumor cell. **D** IF staining for KI67 (red) and counter-staining nuclei with DAPI (blue) in T24 and 5637 cells. **E** Cell cycle analysis was measured by flow cytometry in T24 and 5637 cells. **F** Representative images of SA-β-gal staining for T24 and 5637 cells. **G** The wound healing assay demonstrated the capacity of migration in T24 and 5637 cells. **H** The transwell assay demonstrated the capacity of invasion in T24 and 5637 cells. (The magnification under the microscope is shown as marked in the figure. *p < 0.05, **p < 0.01)

To evaluate the biological function of AURKB in BC, AURKB stable knockdown T24 and 5637 cell lines were constructed. RT-qPCR was used to validate the knockdown transfection efficiency (Fig. 2A). The results of CCK-8 assay demonstrated that AURKB knockdown reduced BC cell proliferation ability in T24 and 5637 cell lines (Fig. 2B). The proliferation ability of a single tumor cell was assessed by the colony-forming assay, and the results were consistent with CCK-8 assay (Fig. 2C). Furthermore, IF staining showed that the proliferation marker KI67 was downregulated in AURKB knockdown BC cell lines (Fig. 2D).

For tumor cell proliferation, cell cycle is an essential process [25]. Therefore, we detected the cell cycle using flow cytometry to investigate whether AURKB knockdown affected cell cycle progression in BC cells. Notably, we found more AURKB knockdown cells than negative control cells in G2/M phase and fewer AURKB knockdown cells in G0/G1 and S phases, indicating the cell cycle was blocked in G2/M phase (Fig. 2E, p < 0.01). Next, we quantified the degree of cellular senescence by SA-β-gal staining, which showed a significant increase in the percentage of SA-β-gal^+^ cells in the AURKB knockdown group (Fig. 2F). Senescent cells produce proinflammatory molecules in what is known as the senescence-associated secretory phenotype (SASP) [26]. To further demonstrate that AURKB is associated with BC cellular senescence, we detected senescence-related molecules and the classic components of SASP using the RT-qPCR. As shown in Additional file 1: Figure S1, AURKB knockdown significantly reduced LaminB1, concomitant with elevated p16, IL-1β, IL-6 and TNF-α. Collectively, these findings suggest that AURKB knockdown suppressed cell proliferation, and contributed to cell cycle arrest and cellular senescence.

Moreover, we detected the migration and invasion of BC cells using the wound healing assay and transwell cell invasion assay. The results demonstrated that AURKB knockdown suppressed migration and invasion in BC cells (Fig. 2G, H). We detected the rate of cell apoptosis using flow cytometry. The results showed that the apoptosis rate was upregulated after AURKB knockdown (Additional file 2: Figure S2). All these findings revealed that AURKB played the protumoural role in BC.

### AURKB interacts with and modulates the expression of MAD2L2

**Fig. 3:**
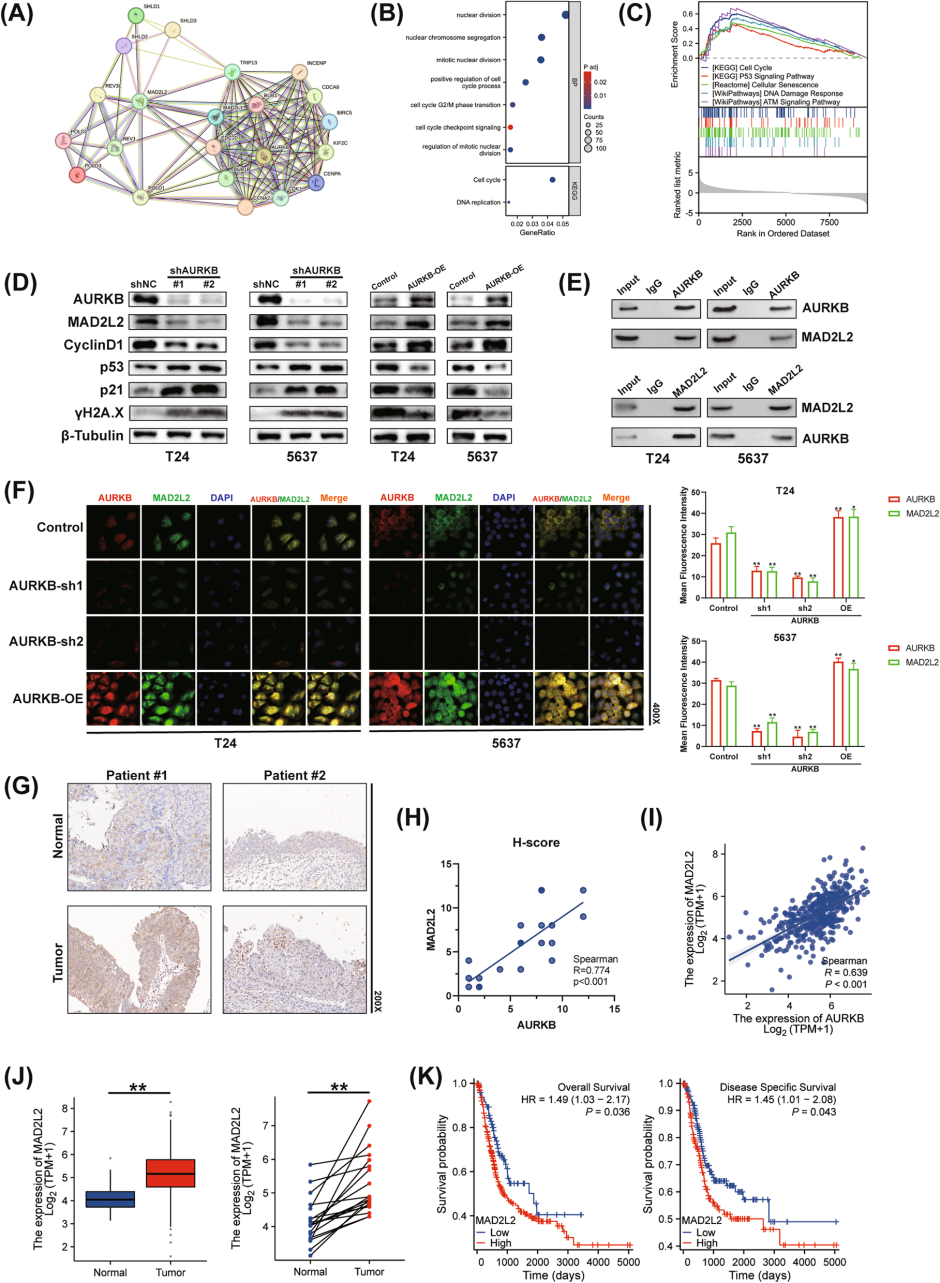
AURKB interacts with and promotes the expression of MAD2L2. **A** Protein interaction network map of proteins bound to AURKB. **B** GO/KEGG enrichment analysis of AURKB in TCGA database. **C** GSEA results of AURKB in TCGA database. **D** Western blot analysis of AURKB, MAD2L2, CyclinD1, p53, p21 and γH2A.X expression. **E** Co-IP indicated the interaction between AURKB and MAD2L2 in BC cells. **F** T24 and 5637 cells were subjected to IF staining for AURKB (red) or MAD2L2 (green) and nuclei with DAPI (blue). **G** Representative IHC images of MAD2L2 in BC tissues and adjacent normal tissues. **H** The correlation analysis of AURKB and MAD2L2 H-score of IHC in patient samples. **I** The correlation analysis between AURKB and MAD2L2 expression in TCGA database. **J** Differential MAD2L2 expression in BC tissues and normal bladder tissues in unpaired and paired samples. **K** Kaplan–Meier survival curves for OS and DSS comparing the high and low expression of MAD2L2 in TCGA database. (The magnification under the microscope is shown as marked in the figure. *p < 0.05, **p < 0.01)

To further investigate the biological functions of AURKB in BC, the STRING database was used to construct a PPI network (Fig. 3A). Next, we performed pathway enrichment analysis for GO and KEGG. The results showed that AURKB plays a vital role in mitotic nuclear division, cell cycle G2/M phase transition and DNA replication (Fig. 3B). Moreover, GSEA was performed to investigate the underlying signaling pathways related to AURKB. According to GSEA findings, gene sets related to cell cycle, the p53 signaling pathway, cellular senescence, DNA damage response, and the ataxia telangiectasia-mutated (ATM) signaling pathway are enriched in the AURKB highly expressed group (Fig. 3C). Next, we detected proteins using western blot analysis. As shown in Fig. 3D, AURKB knockdown significantly decreased the levels of MAD2L2 and Cyclin D1, concomitant with increased expression of p53, p21, and γH2A.X, which is an indicator of DNA damage [27]. Meanwhile, AURKB overexpression produced results opposite to knockdown (Fig. 3D). To further reveal the relationship between AURKB and MAD2L2, Co-IP experiments were performed and the results verified that AURKB can indeed interact with MAD2L2 in BC cells (Fig. 3E). Furthermore, the expression and localization of AURKB and MAD2L2 were observed by IF staining (Fig. 3F). Consistent with western blot analysis, the results suggested that AURKB modulated the expression of MAD2L2. What’s more, AURKB and MAD2L2 were co-localized in both nuclei and cytoplasm. Through IHC analyses, we found that AURKB was significantly correlated with the expression of MAD2L2 in tissues of human specimens (R = 0.774, p < 0.001, Fig. 3G, H). This finding was verified by RNA-seq data from TCGA (R = 0.639, p < 0.001, Fig. 3I). Moreover, similar to AURKB, MAD2L2 was highly expressed in pan-cancer (Additional file 3: Figure S3). MAD2L2 expression was higher in bladder cancer tissues compared to normal tissues in unpaired and paired samples, and high MAD2L2 expression was associated with poor prognosis for BC patients in the TCGA database (Fig. 3J, K). We performed IHC staining for p53 and found the expression of p53 was correlated with the expression of AURKB (R = − 0.591, p < 0.01, Additional file 4: Figure S4) and MAD2L2 (R = − 0.529, p < 0.05, Additional file 4: Figure S4) in tissues of human specimens. Taken together, these findings suggested that AURKB interacted with MAD2L2 and both AURKB and MAD2L2 were correlated with the expression of p53 in BC.

### MAD2L2 overexpression rescues the effect of AURKB knockdown on BC cells

**Fig. 4:**
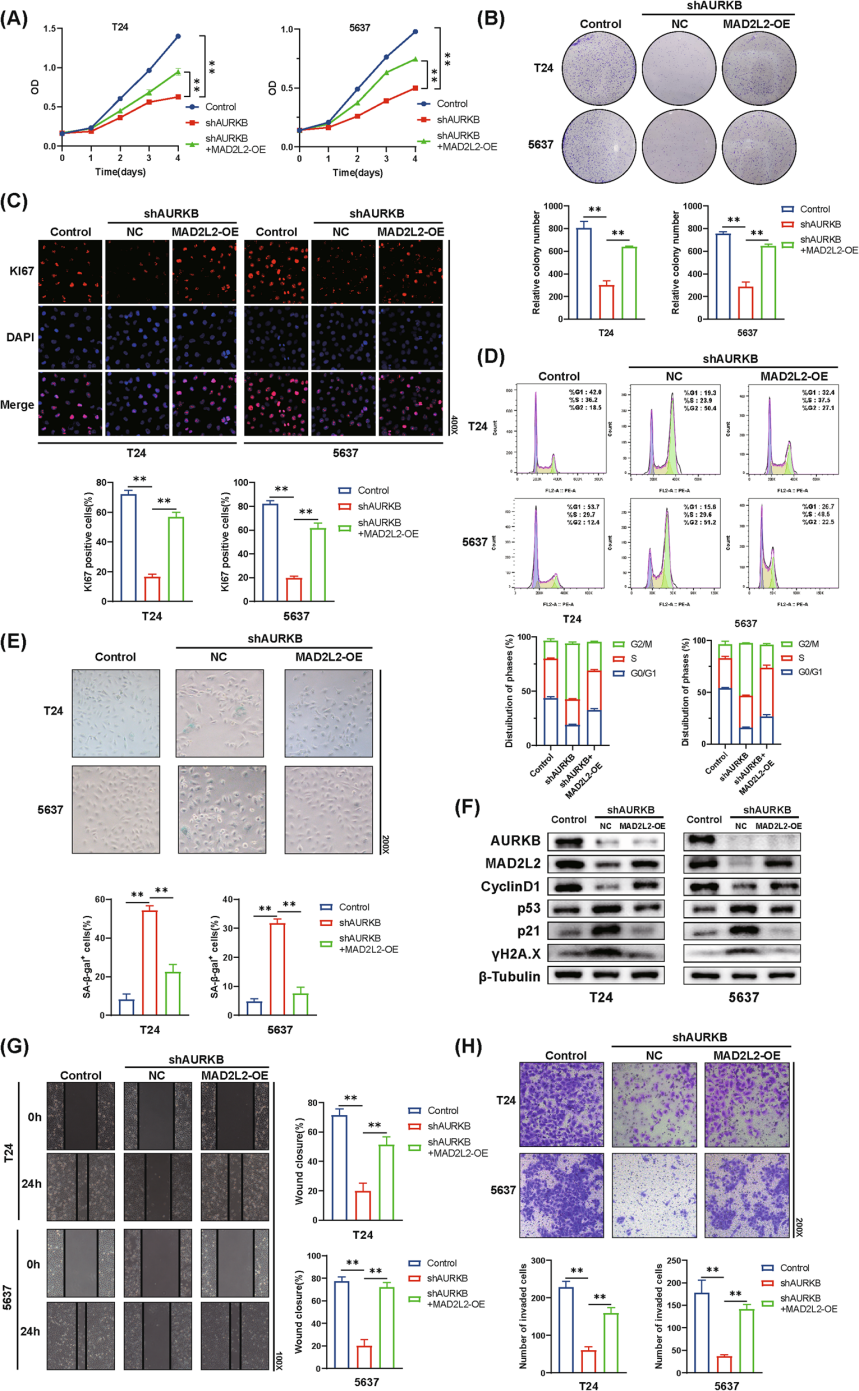
AURKB ablation upregulates the p53 pathway and suppresses BC cell progression via MAD2L2. **A**-**B** Cell growth was detected by CCK-8 assay (**A**) and colony-forming assay (**B**). **C** KI67 detection using IF staining. **D** Cell cycle analysis was measured by flow cytometry. **E** Representative images of SA-β-gal staining. **F** Western blot analysis of AURKB, MAD2L2, CyclinD1, p53, p21 and γH2A.X expression. **G** The wound healing assay demonstrated the capacity of migration. **H** The transwell assay demonstrated the capacity of invasion. (The magnification under the microscope is shown as marked in the figure. *p < 0.05, **p < 0.01)

**Fig. 5:**
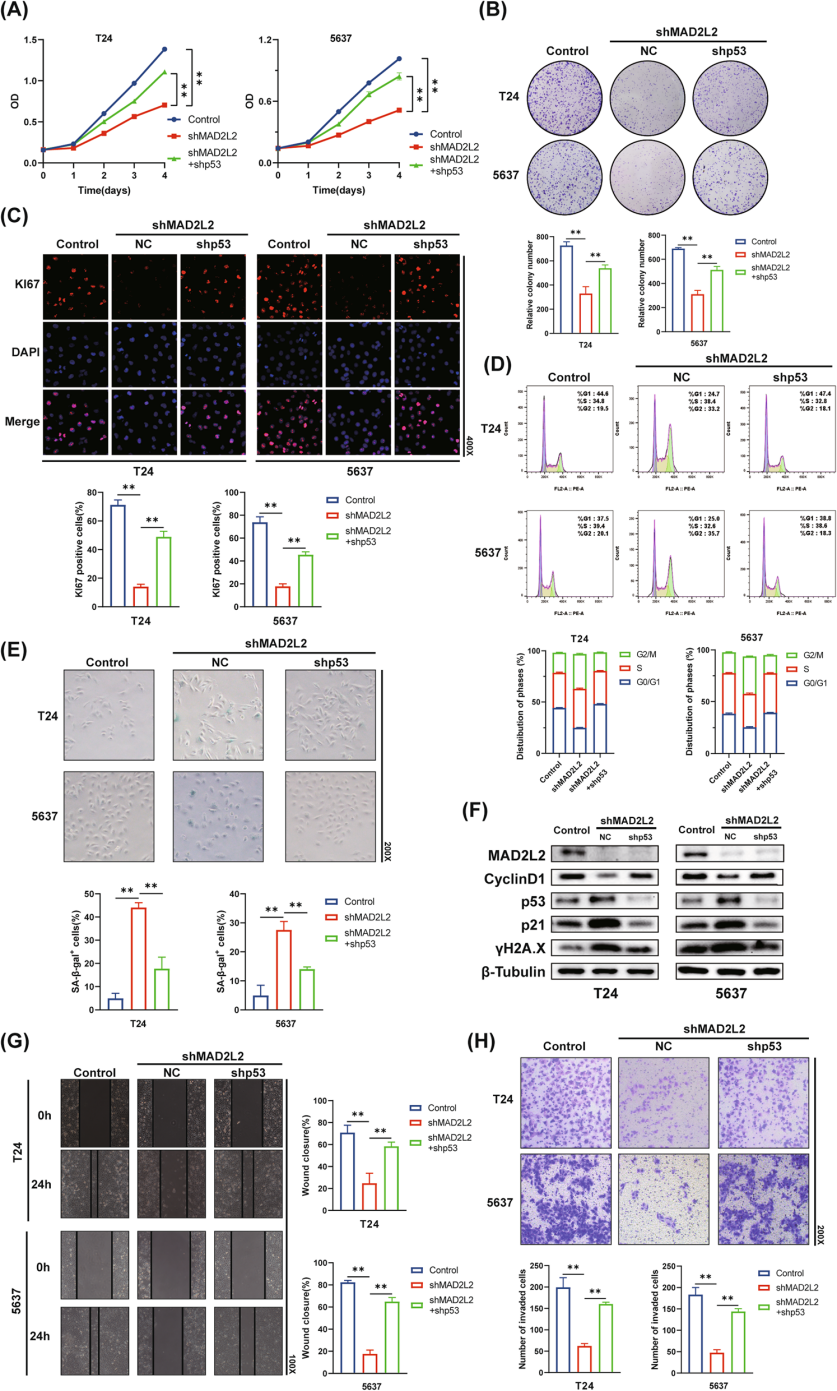
Ablation of MAD2L2 suppresses BC cell progression via p53 DDR pathway. **A**, **B** Cell growth was detected by CCK-8 assay (**A**) and colony-forming assay (**B**). **C** KI67 detection using IF staining. **D** Cell cycle analysis was measured by flow cytometry. **E** Representative images of SA-β-gal staining. **F** Western blot analysis of MAD2L2, CyclinD1, p53, p21 and γH2A.X expression. **G** The wound healing assay for T24 and 5637 cells. **H** The transwell assay for T24 and 5637 cells. (The magnification under the microscope is shown as marked in the figure. *p < 0.05, **p < 0.01)

To investigate whether MAD2L2 plays a role in the suppression of proliferation, cell cycle arrest and cellular senescence induced by knockdown of AURKB, a set of rescuing experiments was performed. The overexpression of MAD2L2 remarkably rescued the proliferation capacity of T24 and 5637 cells through CCK-8 assay, colony-forming assay and IF assay (Fig. 4A–C). Moreover, MAD2L2 overexpression significantly reduced cell cycle G2/M phase arrest and cellular senescence induced by knockdown of AURKB, accompanied with reversed the expression of p16, LaminB1, IL-1β, IL-6 and TNF-α (Fig. 5D, E, and Additional file 1: Figure S1). As expected, western blot analysis revealed that the regulation of AURKB knockdown on p53 pathway, CyclinD1 and γH2A.X, were reversed after MAD2L2 overexpression (Fig. 4F). Furthermore, MAD2L2 promoted the migratory and invasion of BC cells after AURKB knockdown (Fig. 4G, H). Compared with the control group, MAD2L2 overexpression promoted cell proliferation, migration and invasion, reduced the percentage of cells in cell cycle G2/M phase and cellular senescence (Additional file 5: Figure S5A–H). Overall, these findings suggested that knockdown of AURKB downregulated MAD2L2 to upregulate the p53 pathway, cell cycle arrest and cellular senescence, resulting in the suppression of BC cell proliferation.

### Ablation of MAD2L2 leads to cell cycle arrest and cellular senescence via p53 DDR pathway, resulting in the suppression of cell proliferation of BC cells

So far, we have revealed knockdown of AURKB significantly upregulated the expression of p53 and p21, which was reversed by overexpression of MAD2L2. To further investigate the role of MAD2L2 in p53 DDR pathway and cell proliferation of BC cells, we employed shRNAs to effectively ablate MAD2L2 or p53. Similar to the knockdown of AURKB, ablation of MAD2L2 inhibited T24 and 5637 cells proliferation and viability (Fig. 5A–C), concomitant with the induction of cell cycle G2/M phase arrest and cellular senescence (Fig. 5D and E). However, silencing of p53 significantly blocked the effect of MAD2L2 knockdown on BC cells (Fig. 5A–E). Additionally, as shown in Fig. 5F and Additional file 6: Figure S6, ablation of p53 completely blocked the MAD2L2 knockdown-induced upregulation of p21, γH2A.X and SASP and downregulation of CyclinD1 and LaminB1. Notably, p16, another senescence-related pathway, was not downregulated after ablation of p53. The wound healing assay and transwell cell invasion assay were performed one more time, and the results showed that ablation of p53 rescued the migratory and invasion of BC cells (Fig. 5G and H). Together, these findings revealed that MAD2L2 knockdown-induced suppression of cell proliferation was dependent on p53 DDR pathway.

### Effect of AURKB and MAD2L2 on BC growth in vivo

**Fig. 6:**
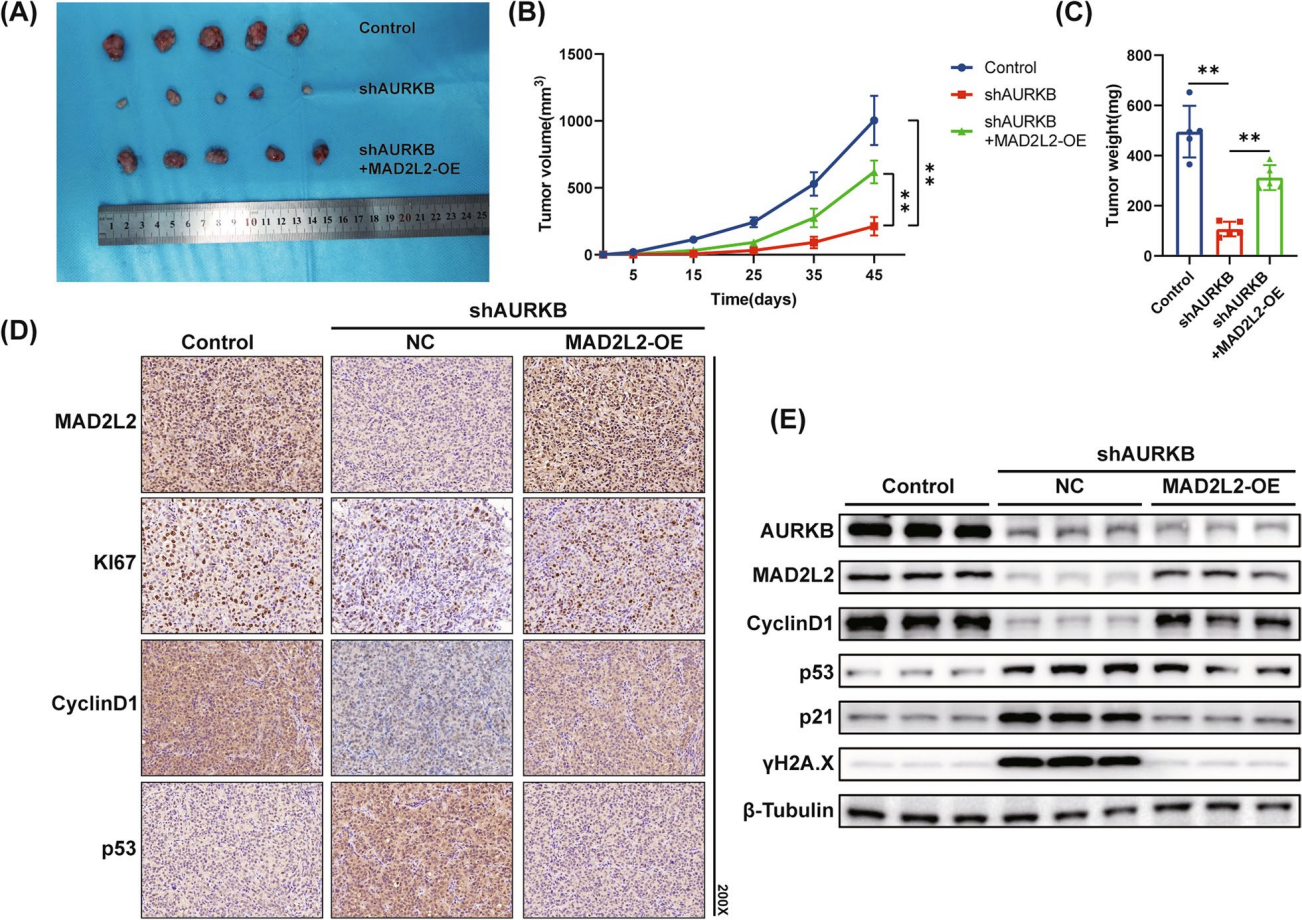
AURKB promotes BC growth and downregulats p53 DDR pahway by regulating MAD2L2 expression in vivo. **A** Images of dissected T24 xenograft tumors. **B**, **C** Tumor volume and weight in each group.**D **Representative images of IHC staining for MAD2L2, KI67, CyclinD1 and p53 in each group. **E** Western blot analysis of AURKB, MAD2L2, CyclinD1, p53, p21 and γH2A.X expression in each group (The magnification under the microscope is shown as marked in the figure. *p < 0.05, **p < 0.01)

To further explore the function of AURKB and MAD2L2 in vivo, we established a T24 xenograft tumor model in nude mice. As the results showed, we found that the growth of tumor cell xenografts was significantly suppressed in the AURKB knockdown group, concomitant with the reduction of the tumor weight. However, MAD2L2 overexpression significantly reversed the effect caused by AURKB (Fig. 6A–C). The IHC staining indicated that MAD2L2, KI67 and CyclinD1 expression was downregulated and p53 expression was upregulated in the AURKB knockdown group. What's more, MAD2L2 overexpression group rescued all the results caused by AURKB knockdown (Fig. 6D). Simultaneously, the lysates of pooled tumor tissues in nude mice were subjected to western blot analysis (Fig. 6E). As expected, the results were consistent with our previous in vitro results. Taken together, AURKB promoted BC growth and downregulated p53 DDR pathway by regulating MAD2L2 expression in vivo.

## Discussion

Bladder cancer (BC) is characterized by a high recurrence rate and a variable rate of progression [28]. Recent studies have focused on the identification of novel molecular targets and treatment options to prevent the recurrence and progression of bladder cancer. Previous studies have indicated that AURKB is overexpressed in various tumors and contributes to tumor development and progression [11]. Thus, AURKB has emerged as an important drug target. However, the molecular mechanism underlying the action of AURKB in BC progression remains unclear.

Genomic instability is a common hallmark of cancer [29]; DDR represents a series of signaling pathways that repair DNA damage and promote the maintenance of genome integrity [30]. Defects in the DDR are associated with neoplastic transformation and proliferation [31]. Therefore, the DDR is regarded as an emerging therapeutic target in cancer. Various processes, such as cell proliferation, cell cycle arrest, and cellular senescence, are influenced by the DDR pathway [32]. Moreover, DDR is regulated by AURKB in multiple types of cancers. AURKB has been shown to play a role in tumorigenesis through stabilizing MYC oncogene [15], activating the AKT/GSK3β pathway [33], and accelerating p53 degradation [23, 34]. In the present study, we found that AURKB was significantly upregulated, and its levels correlated with a poor prognosis in patients with BC. Knockdown of AURKB suppressed cell proliferation, cell cycle progression, migration, and invasion and upregulated cellular senescence. In agreement with previous studies, our results suggest that AURKB promotes BC progression by regulating the DDR pathway, which merits further investigation.

MAD2L2 is a regulatory subunit of DNA polymerase ζ and plays a critical role in DNA repair [24]. MAD2L2 and AURKB are frequently overexpressed in human tumors, leading to chromosomal instability and tumorigenesis [35]. However, the synergistic oncogenic roles of AURKB and MAD2L2 in mitotic DDR remain unclear. In addition to its roles in mitotic control and the DDR pathway, MAD2L2 affects cancer development in various ways, such as regulating TCF4-induced cancer metastasis [36, 37], inhibiting colorectal cancer growth by promoting NCOA3 degradation [37], and recruiting PRDX2 to confer radioresistance in esophageal squamous cell carcinoma [38]. In the present study, we found that AURKB interacts and colocalizes with MAD2L2 in BC cells. Furthermore, MAD2L2 overexpression markedly rescued BC cell proliferation suppressed by AURKB knockdown in vitro and in vivo, accompanied by a significant suppression of cell cycle arrest in the G2/M phase and cellular senescence. Migration and invasion of BC cells were rescued by MAD2L2. These findings indicated that MAD2L2 plays an important role in the promotion of BC by AURKB.

As a critical tumor suppressor gene that is mutated in more than 50% of all human cancers, p53 is an attractive therapeutic target for treating cancer [39]. Further, p53 plays an important role in controlling cellular proliferation in the context of DNA damage [40]. The p53 pathway is activated by the ATM pathway, which is the primary detector of DNA damage [21]. AURKB has been shown to be significantly associated with the p53 pathway [41, 42], which is confirmed by results of this study. However, few studies have investigated the roles of AURKB and MAD2L2 in the p53 DDR pathway in BC. In the present study, we found that p53 expression was upregulated by knockdown of AURKB and MAD2L2 and downregulated by overexpression of AURKB and MAD2L2. To obtain sufficient evidence, we detected SASP, the expression of proteins related to the cellular senescence pathway, and an indicator of DNA damage. These results are consistent with the findings for p53 expression. To investigate whether the p53 DDR pathway plays a role in the suppression of BC cell proliferation induced by MAD2L2 knockdown, we employed shRNA was used to effectively ablate p53 expression. The results showed that ablation of p53 remarkably rescued the effect of MAD2L2 knockdown in BC cells. These findings further confirmed that MAD2L2 downregulates p53 to overcome p53-induced cell cycle arrest and cellular senescence, resulting in the promotion of the proliferation, migration, and invasion abilities of BC cells.

Chemotherapy-induced drug resistance is a challenging issue in cancer treatment. Research on DDR and genomic instability is crucial to find new therapeutic options. The combination of DDR inhibitors and other drugs targeting DDR proteins can block multiple pathways on which cancer cells rely for survival and may also be used to sensitize cancer cells to radiotherapy or chemotherapy [31]. Our results suggest that AURKB activates MAD2L2 expression to downregulate the p53 DDR pathway, thereby promoting BC progression. Based on the identified molecular mechanisms, AURKB could be identified as a novel target for DDR-targeting therapy in BC. A variety of small-molecule inhibitors targeting AURKs have been developed and are currently in different phases of clinical trials [12]. The present study supports the development of AURKB inhibitors that are either effective against DDR molecules or that inhibit their interactions.

This study has a few limitations that need to be addressed. Although we found that AURKB interacts with MAD2L2, the mechanism by which these two molecules interact requires further investigation. Secondly, the specific mechanisms by which AURKB promotes the growth of BC cells via the MAD2L2/p53 DDR pathway in vivo have not been explored. This is a future direction for our research.

## Conclusions

The present study suggests that AURKB is highly expressed in patients with BC and promotes the occurrence of malignant phenotypes in vitro and in vivo. AURKB activates MAD2L2 expression to downregulate the p53 DDR pathway, resulting in the promotion of BC cell proliferation, cell cycle progression, and migration and invasion abilities of BC cells, and the inhibition of cellular senescence. Thus, AURKB may serve as a potential molecular marker and a novel anticancer therapeutic target for BC.

### Supplementary Information

**Additional file 1: Figure S1.** The RT-qPCR detected the mRNA levels of p16, LaminB1, IL-1β, IL-6 and TNF-α. (**p < 0.01).

**Additional file 2: Figure S2.** Cell apoptosis was measured by flow cytometry for T24 and 5637 cells. (**p < 0.01).

**Additional file 3: Figure S3.** Differential expression of MTHFD2 in various types of cancer analyzed by TIMER. (**p < 0.01, *** p < 0.001).

**Additional file 4: Figure S4.** Representative IHC images of p53 and the correlation analysis of p53 and AURKB/MAD2L2 H-score in BC patient samples. (The magnification under the microscope is shown as marked in the figure).

**Additional file 5: Figure S5.** MAD2L2 overexpression promoted cell proliferation, migration and invasion, and reduced cellular senescence. (A) Western blot analysis after MAD2L2 overexpression. (B-C) Cell growth was detected by CCK-8 assay (B) and colony-forming assay (C). (D) KI67 detection using IF staining. (E) Cell cycle analysis was measured by flow cytometry. (F) Representative images of SA-β-gal staining. (G) The wound healing assay for T24 and 5637 cells. (H) The transwell assay for T24 and 5637 cells. (The magnification under the microscope is shown as marked in the figure. *p < 0.05, **p < 0.01).

**Additional file 6: Figure S6.** The RT-qPCR detected the mRNA levels of p16, LaminB1, IL-1β, IL-6 and TNF-α. (*p < 0.05, **p < 0.01).

**Additional file 7: Table S1.** Primers were used in this study.

**Additional file 8.** Method details.

## Data Availability

The datasets used and/or analysed during the current study are available from the authors on reasonable request.

## Abbreviations

BC: Bladder cancer
NMIBC: Non-muscle-invasive bladder cancer
MIBC: Muscle-invasive bladder cancer
BCG: Bacillus Calmette-Guerin
AURKB: Aurora kinase B
MAD2L2: Mitotic arrest-deficient 2-like protein 2
DDR: DNA damage response
DSBs: DNA double-strand breaks
TCGA: The Cancer Genome Atlas
GEO: Gene Expression Omnibus
TIMER: Tumor immune estimation resource
PPI: Protein–protein interaction
GSEA: Gene set enrichment analysis
FDR: False discovery rate
RT-qPCR: Quantitative reverse transcription polymerase chain reaction
IF: Immunofluorescence
IHC: Immunohistochemistry
Co-IP: Co-immunoprecipitation
SA-β-gal: Senescence-associated β-galactosidase
OS: Overall survival
DSS: Disease specific survival
SASP: Senescence-associated secretory phenotype
ATM: Ataxia telangiectasia-mutated
