# Supplementary figures and images for: AURKB promotes bladder cancer progression by deregulating the p53 DNA damage response pathway via MAD2L2
Source: J Transl Med. 2024 Mar 21;22:295. doi: 10.1186/s12967-024-05099-6 (PMC10956193; doi:10.1186/s12967-024-05099-6)

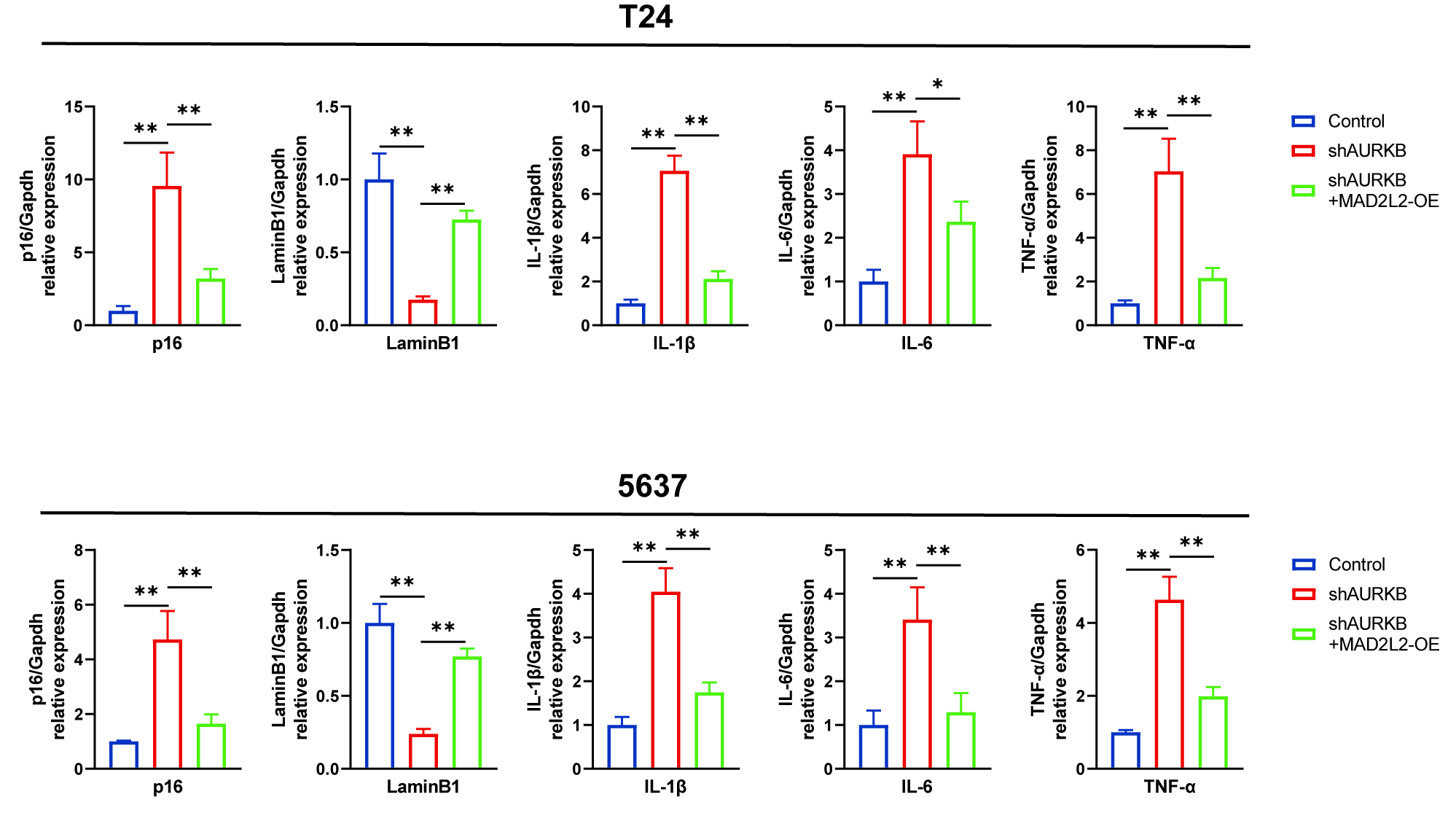

Supplement: Supplementary file 1 — Additional file 1: Figure S1. The RT-qPCR detected the mRNA levels of p16, LaminB1, IL-1β, IL-6 and TNF-α. (**p < 0.01). [file 12967_2024_5099_MOESM1_ESM.tif]

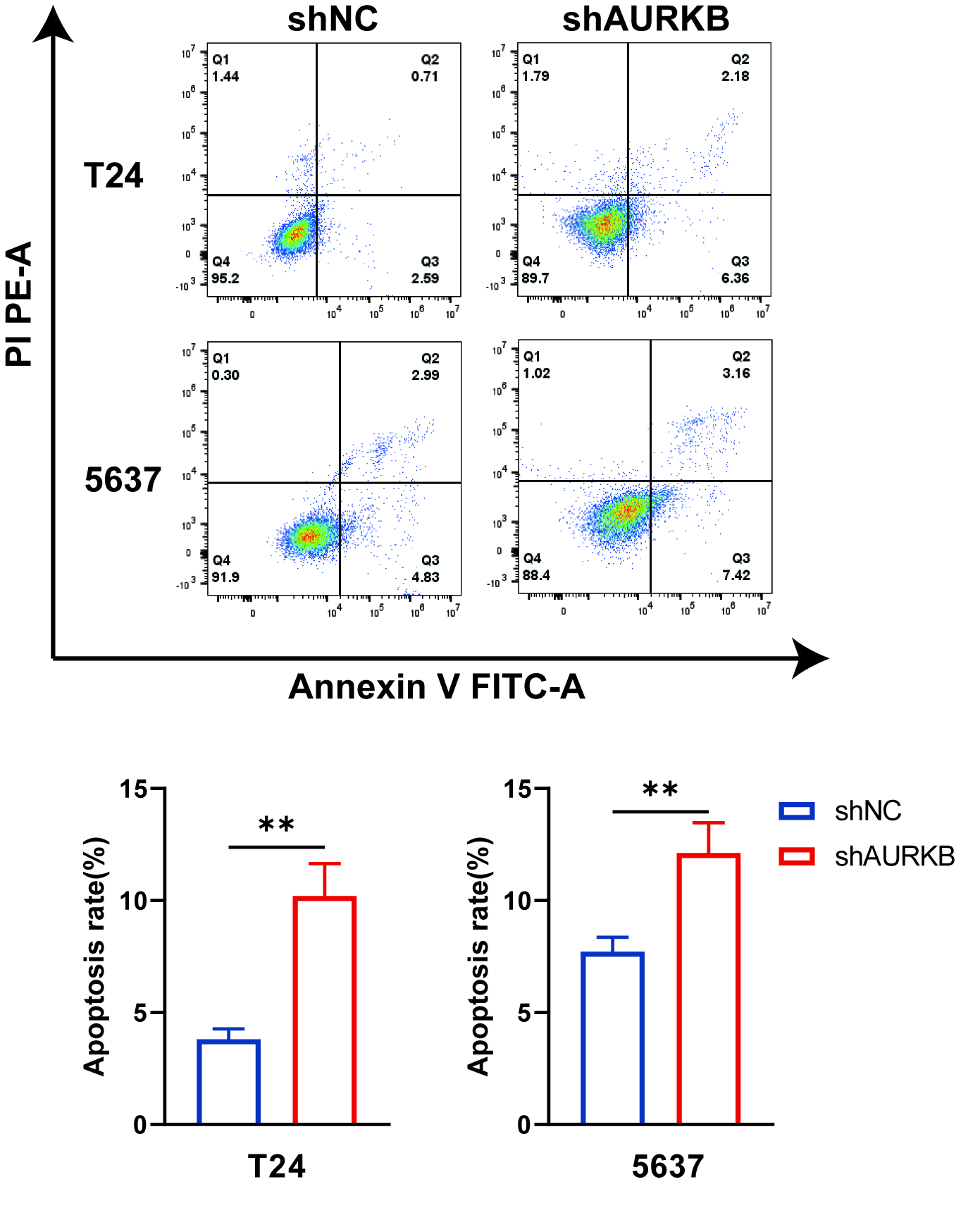

Supplement: Supplementary file 2 — Additional file 2: Figure S2. Cell apoptosis was measured by flow cytometry for T24 and 5637 cells. (**p < 0.01). [file 12967_2024_5099_MOESM2_ESM.tif]

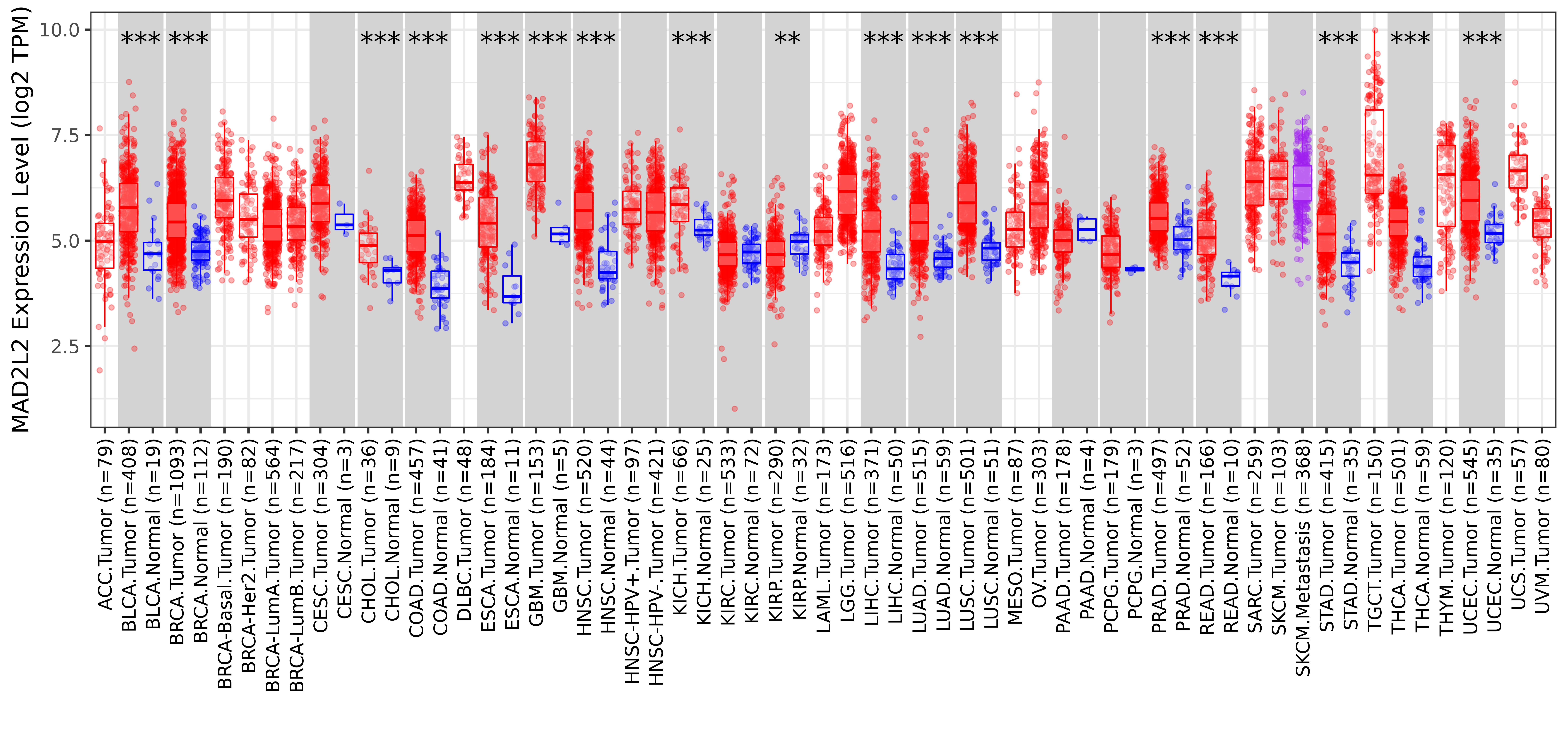

Supplement: Supplementary file 3 — Additional file 3: Figure S3. Differential expression of MTHFD2 in various types of cancer analyzed by TIMER. (**p < 0.01, *** p < 0.001). [file 12967_2024_5099_MOESM3_ESM.jpg]

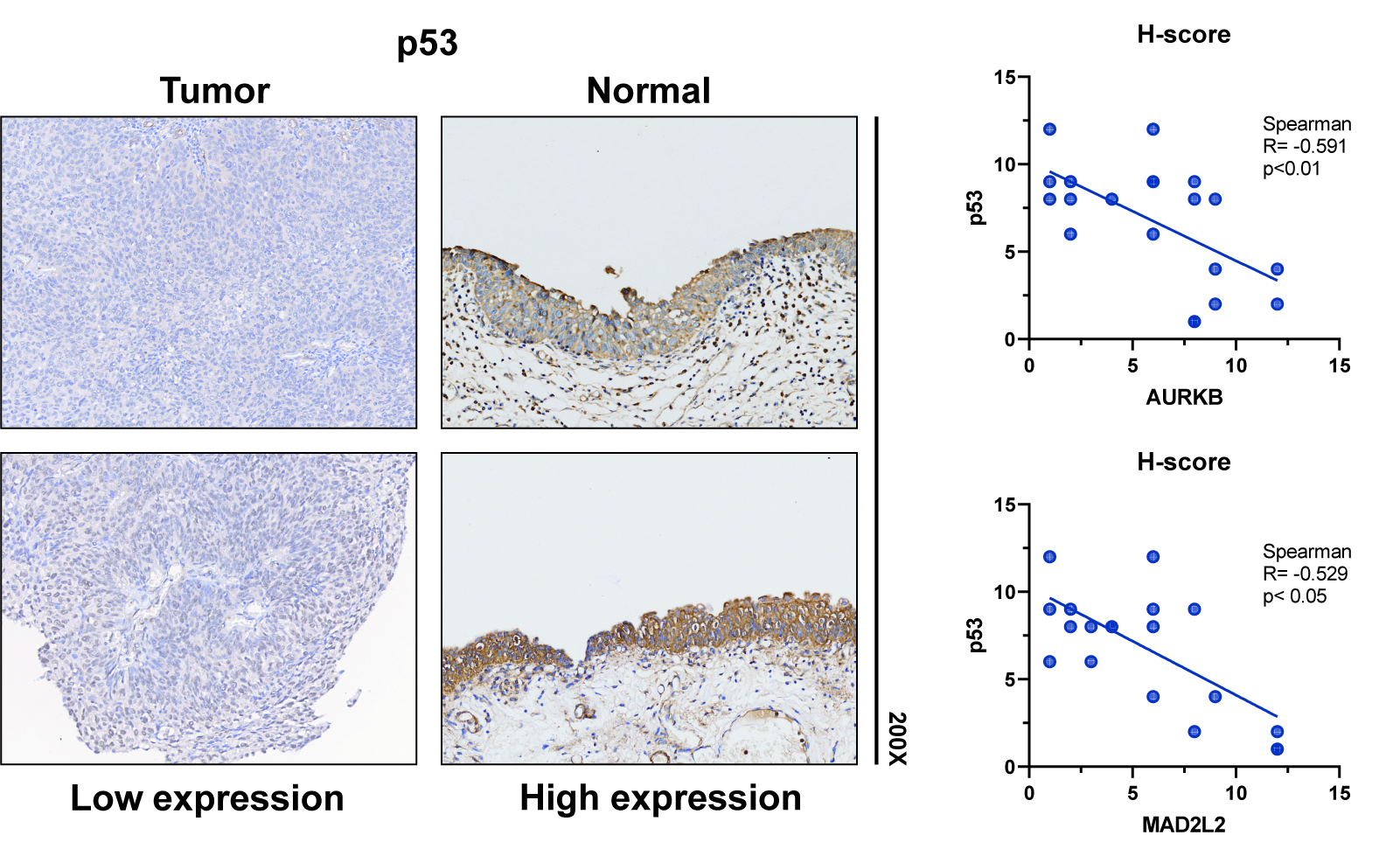

Supplement: Supplementary file 4 — Additional file 4: Figure S4. Representative IHC images of p53 and the correlation analysis of p53 and AURKB/MAD2L2 H-score in BC patient samples. (The magnification under the microscope is shown as marked in the figure). [file 12967_2024_5099_MOESM4_ESM.tif]

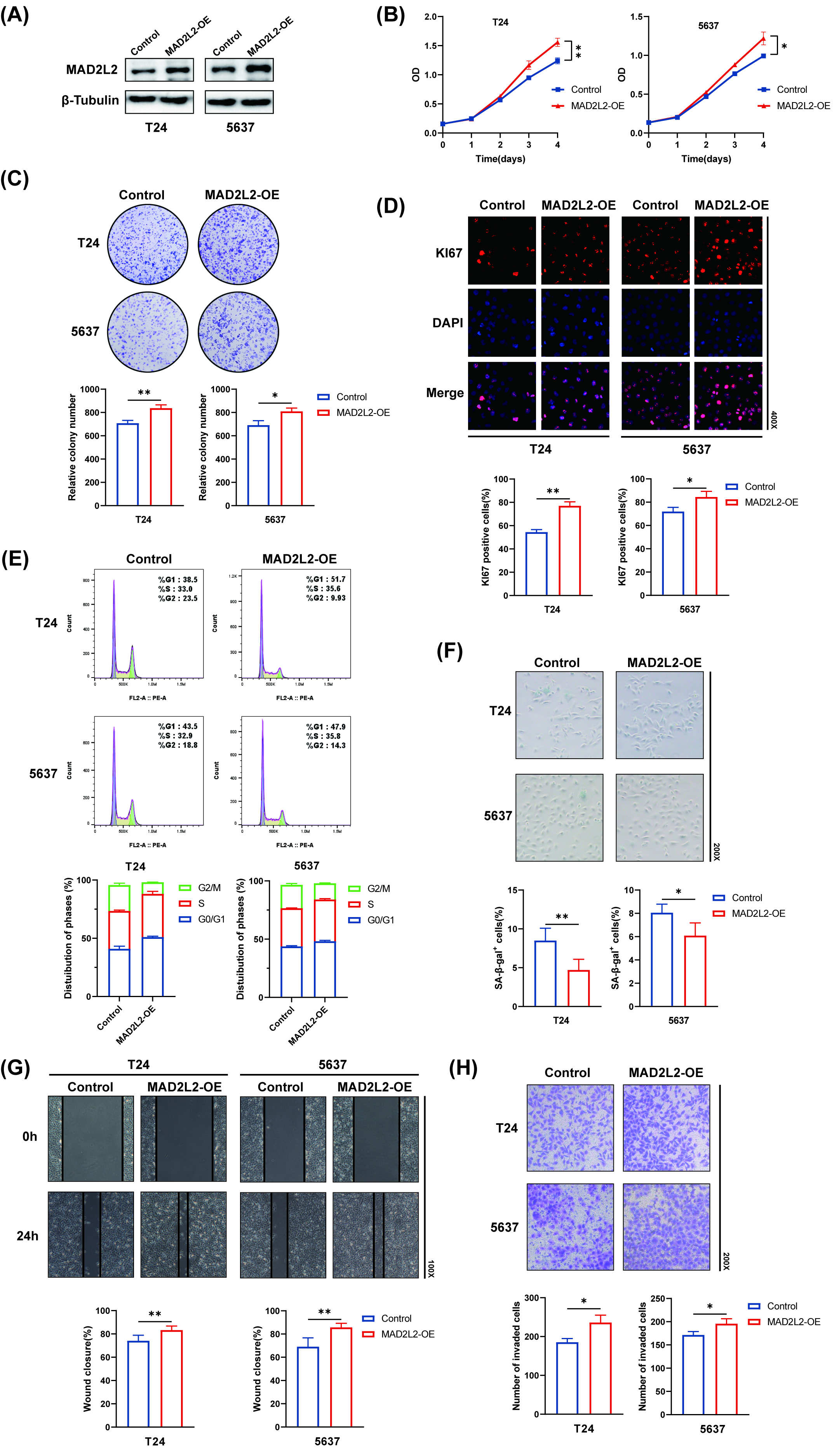

Supplement: Supplementary file 5 — Additional file 5: Figure S5. MAD2L2 overexpression promoted cell proliferation, migration and invasion, and reduced cellular senescence. (A) Western blot analysis after MAD2L2 overexpression. (B-C) Cell growth was detected by CCK-8 assay (B) and colony-forming assay (C). (D) KI67 detection using IF staining. (E) Cell cycle analysis was measured by flow cytometry. (F) Representative images of SA-β-gal staining. (G) The wound healing assay for T24 and 5637 cells. (H) The transwell assay for T24 and 5637 cells. (The magnification under the microscope is shown as marked in the figure. *p < 0.05, **p < 0.01). [file 12967_2024_5099_MOESM5_ESM.tif]

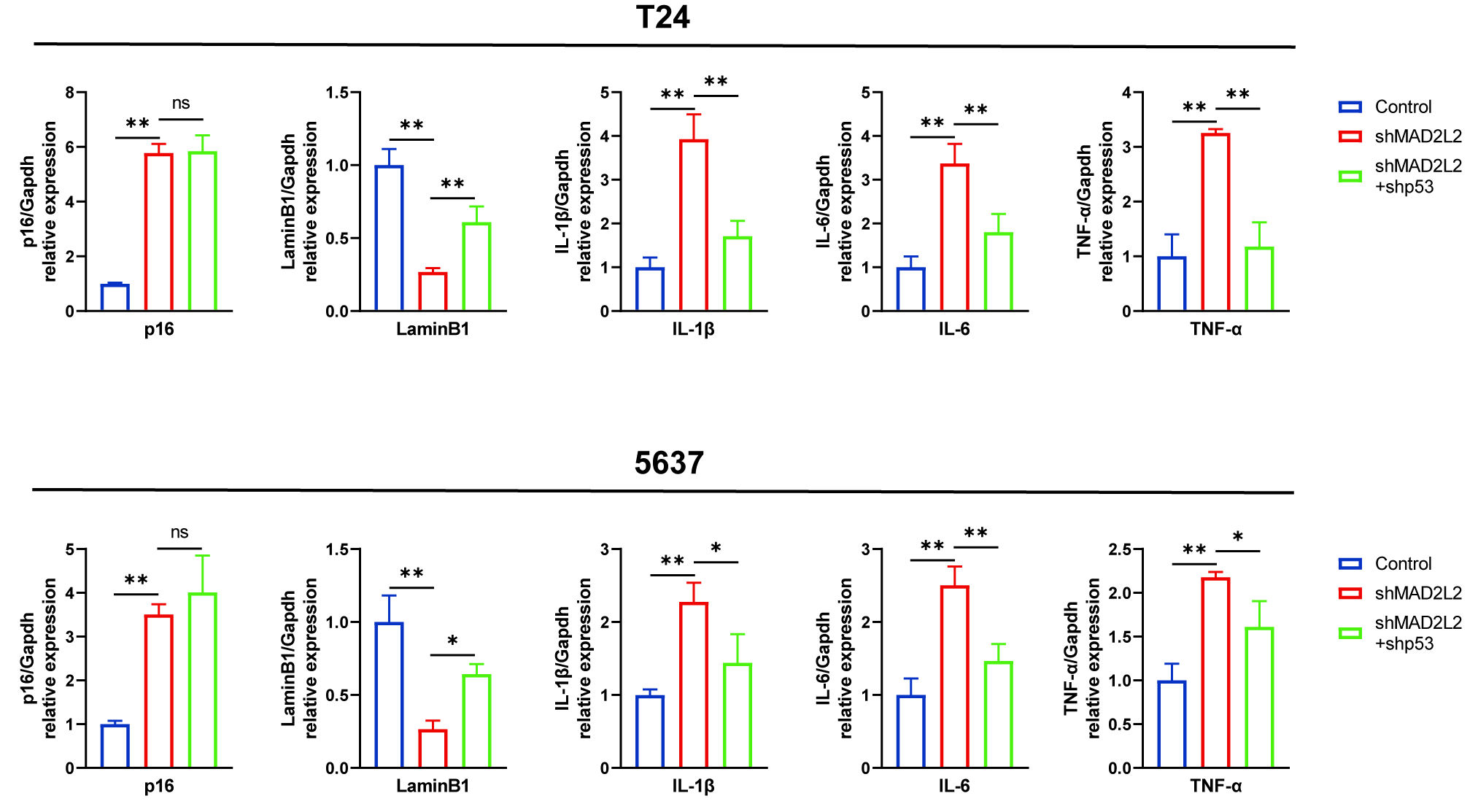

Supplement: Supplementary file 6 — Additional file 6: Figure S6. The RT-qPCR detected the mRNA levels of p16, LaminB1, IL-1β, IL-6 and TNF-α. (*p < 0.05, **p < 0.01). [file 12967_2024_5099_MOESM6_ESM.tif]
