# Supplementary material for: AURKB promotes bladder cancer progression by deregulating the p53 DNA damage response pathway via MAD2L2
Source: J Transl Med. 2024 Mar 21;22:295. doi: 10.1186/s12967-024-05099-6 (PMC10956193; doi:10.1186/s12967-024-05099-6)
Supplement: Supplementary file 7 — Additional file 7: Table S1. Primers were used in this study. [file 12967_2024_5099_MOESM7_ESM.docx]

**Table S1. Primers were used in this study.**

| Target | Sequence |
| --- | --- |
| *AURKB* F | GCAGCGAACAGCCACGATC |
| *AURKB* R | GACCAGCCGAAGTCAGCAATC |
| p16 F | GCCCAACGCACCGAATAGTTAC |
| p16 R | GCAGCAGCTCCGCCACTC |
| LaminB1 F | GGAGAATCGTTGTCAGAGCCTTAC |
| LaminB1 R | ATGCTTCCTTCTGGTCTCGTTAATC |
| IL-1β F | TGGCTTATTACAGTGGCAATGAGG |
| IL-1β R | AGTGGTGGTCGGAGATTCGTAG |
| IL-6 F | TTCGGTCCAGTTGCCTTCTCC |
| IL-6 R | TTCTGAAGAGGTGAGTGGCTGTC |
| TNF-α F | CTCATCTACTCCCAGGTCCTCTTC |
| TNF-α R | CGATGCGGCTGATGGTGTG |
| GAPDH F | CCCGTGGAATGGAATGAGATT |
| GAPDH R | CTCAGGAGAGGAGCCATTT |
