# Supplementary material for: AURKB promotes bladder cancer progression by deregulating the p53 DNA damage response pathway via MAD2L2
Source: J Transl Med. 2024 Mar 21;22:295. doi: 10.1186/s12967-024-05099-6 (PMC10956193; doi:10.1186/s12967-024-05099-6)
Supplement: Supplementary file 8 — Additional file 8. Method details. [file 12967_2024_5099_MOESM8_ESM.docx]

**Additional Methods**

1. **Data mining**

The gene expression RNA sequencing data and corresponding clinical data were downloaded from The Cancer Genome Atlas (TCGA) database (https://portal.gdc.cancer.gov/). Datasets of the GSE13507 cohort were downloaded from the Gene Expression Omnibus (GEO) database (https://www.ncbi.nlm.nih.gov/geo).

1. **Bioinformatics analyses**

The level of AURKB in pan-cancer was analyzed by tumor immune estimation resource (TIMER) 2.0 database (<http://timer.cistrome.org/>). The FPKM data from TCGA and GEO databases were transformed using log2 (TPM + 1) for further calculation. R 4.2.1 was used for further data processing and statistical analysis. We used stats and car packages to analyze the expression of AURKB and MAD2L2 in BC. ROC analysis was performed using the pROC package. To verify the prognostic value of AURKB and MAD2L2, we used the survival package to conduct Kaplan–Meier univariate survival analysis based on the data in GEO and TCGA databases. The protein-protein interaction (PPI) network was constructed using STRING (https://string-db.org/) for searching a database of interacting genes. Based on the AURKB RNA sequencing data of the TCGA, the patients were divided into a high expression group and a low expression group, and the DESeq2 package was used to screen differentially expressed molecules. GO/KEGG term enrichment analyses were conducted using the clusterProfiler package. Gene set enrichment analysis (GSEA) was performed using the C2.cp.kegg.v2022.1.Hs.symbols.gmt with GSEA 4.3.0 software. All results were visualized with the ggplot2 package. In this study, we considered gene sets with nominal p-value < 0.05 and a false discovery rate (FDR) < 0.25 to be statistically significant.

1. **Cell culture**

The human BC cell lines (T24 and 5637) were acquired from the Chinese Academy of Sciences (Shanghai, China). T24 and 5637 were cultured in RPMI-1640 medium (Pricella, Wuhan, China). All culture media contained 10% fetal bovine serum (Pricella) and 1% penicillin/streptomycin (Pricella). Cells were maintained at 37 °C in a humidified 5% CO2 incubator.

- 1. **Cell Resuscitation**

Cryopreserved cells from the liquid nitrogen tanks were quickly thawed in a 37 °C water bath. Following centrifugation at 1000rpm for 5 min, the cell pellets were resuspended in the culture medium. Cells were centrifuged and resuspended in the culture medium again, transferred to cell culture flasks, and cultured in a humidified 5% CO2 incubator at 37 °C. The medium was refreshed on the next day.

- 1. **Cell subculture**

Cells were subcultured when confluency reached approximately 90%. The medium was removed and the cells were gently washed with PBS. Cells were trypsinized using Trypsin-EDTA (0.25%) (Pricella) at 37 °C. The digestion was terminated by the complete medium which contains the fetal bovine serum. Cells adhering to the walls of the cell culture flask were gently blown off using pipette tips, and the cell suspension was transferred to a centrifuge tube. Following centrifugation at 1000rpm for 5 min, the cell pellets were resuspended in the culture medium. Cells were then distributed to other cell culture flasks, and cultured in a humidified 5% CO2 incubator at 37 °C.

- 1. **Cell cryopreservation**

After digestion, cells were centrifuged and resuspended in a commercial cryoprotectant (Pricella). The cell suspension was transferred to sterile cell cryopreservation tubes. The tubes were placed in the refrigerator at -80 °C for 24 h and transferred to liquid nitrogen tanks for long-term storage.

1. **Lentiviral transfection**

For stable knockdown or overexpression of AURKB, MAD2L2 and p53, lentiviral-based shRNAs were synthesized by OBiO Co., Ltd (Shanghai, China). Cells ready for transfection were seeded into 6-well dishes at a density of 1 × 10^6^ cells/well after reaching 40% confluence. Lentivirus (volume = multiplicity of infection (MOI= 10) × cells number/lentivirus titer) mixed with 5 µg/ml polybrene was added to cells. After transfection for 24 h, the medium was refreshed. After transfection for 2-4 days, cells were selected in 4 µg/ml puromycin. The knockdown or overexpression efficiency was validated by Real-Time Quantitative PCR and western blot analysis.

1. **Collection of patient samples**

The neighboring normal tissues and the tumor tissues were sourced from 10 BC patients undergoing cystectomy at Renmin Hospital of Wuhan University. Patient samples were histologically proven BC. The Ethics Committee of Medical School of Wuhan University approved this study. All patients participating in the study obtained informed consent.

The tissue samples were dissected into two parts. One part was immediately stored in liquid nitrogen for RNA extraction, and the other part was fixed in 4% paraformaldehyde for immunohistochemistry.

1. **Quantitative reverse transcription polymerase chain reaction (****RT-qPCR)**
   1. **RNA extraction**

The total RNA from the patient samples and cells was extracted using TRIzol reagent (Servicebio, Wuhan, China). TRIzol-extracted samples were mixed 5:1 with chloroform, shaken vigorously for 20 s, and incubated at room temperature for 10 min. Following centrifugation at 12000×g at 4°C for 15 min, the supernatant was carefully collected and mixed 1:1 with isopropanol. After incubation at RT for 10 min, tubes were centrifuged at 12000×g at 4°C for 15 min. After removing the supernatant, the pellets were washed in cold 75% ethanol. Following centrifugation again, the supernatant was removed and dried pellets were resuspended in DEPC water.

- 1. **RT-PCR**

Total RNA concentrations were measured by NanoDrop 2000 (Thermo Fisher, USA). cDNA was generated using the SweScript All-in-One First-Strand cDNA Synthesis SuperMix for qPCR (Servicebio) with 100ng of RNA as template. Reverse transcription reaction conditions were 25 °C for 5 min, 42 °C for 30 min, 85 °C for 5 s, and 4°C hold. Resulting cDNAs were prepared for RT-qPCR.

- 1. **RT-qPCR**

RT-qPCR was performed with 2 × SYBR Green qPCR Master Mix (Servicebio) using the Lightcycler 4800II (Roche, Basel, Switzerland) following the manufacturer’s instructions. RT-qPCR reactions were performed using the following protocol: Initial denaturation: 95 °C for 30 s. Amplification: 40 cycles of 95 °C for 15 s, 60 °C for 30 s. Recording melting curves. The relative gene expression was calculated using the 2−ΔΔCt method and normalized to the expression of GAPDH. The specific RT-qPCR primer sequences were listed in Supplementary Table S1.

1. **Western blot analysis**

For protein extraction, cells were washed twice with PBS and lysed on ice for 20 min in lysis buffer (RIPA buffer (Servicebio) containing 0.1 mM PMSF (Servicebio) and phosphatase inhibitors (Servicebio)). Tissue protein extraction was prepared by mechanical homogenization of tissue in liquid nitrogen followed by solubilization on ice in lysis buffer. After lysis, all protein extracts were sonicated followed by centrifugation at 12000 ×g at 4  °C for 15 min. The supernatant was collected, and protein concentration was measured by BCA kit (Servicebio). Equal amounts of protein samples were fractionated by SDS/PAGE and transferred to the PVDF membrane. After blocking with a protein-free fast blocking buffer (Servicebio), the membranes were incubated with primary antibodies overnight at 4 °C, followed by washing 3 times with TBST (Tris-buffered saline containing 0.1% Tween 20 (Servicebio)) for 5min. The membranes were incubated with HRP-conjugated secondary antibodies (Servicebio) at room temperature for 1 h. After washing 3 times with TBST for 5min, all bands were measured using an ECL kit (Servicebio) by chemiluminescence (Bio-Rad) and analyzed using ImageJ software. The following primary antibodies were used in the present study: AURKB (1:1000, A1020, ABclonal, Wuhan, China), MAD2L2 (1:1000, A4630, ABclonal), Cyclin D1 (1:1000, A19038, ABclonal), p53 (1:1000, ab32049, Abcam, UK), p21 (1:1000, A1483, ABclonal), γH2A.X (1:5000, T56572, Abmart, Shanghai, China), β-Tubulin (1:5000, M20005, Abmart).

1. **Immunofluorescence (IF)**

For IF staining, cells were fixed with 4% paraformaldehyde for 15 min at room temperature, permeabilized with 0.5% TritonX-100 for 15 min, washed 3 times with PBS, blocked with 5% BSA for 1 h, and incubated with primary antibodies overnight at 4°C in a humidified box. After rinsing 3 times with PBS, cells were further incubated with fluorescence-conjugated secondary antibodies at room temperature for 1 h in the dark. Finally, nuclei were stained with DAPI. Fluorescence images were acquired using a fluorescence microscope (BX53, Olympus, Japan). Antibodies used in IF were as follows: KI67 (1:200, 27309-1-AP, Proteintech, Wuhan, China), AURKB (1:50, A19539, ABclonal), MAD2L2 (1:50, A4630, ABclonal).

1. **Immunohistochemistry (IHC) staining**

Tissues from patients and nude mice were embedded in paraffin and sliced into 5 µm thickness. The tissue sections were dewaxed with xylene, and hydrated with decreasing concentrations of ethanol (100%, 95% and 75%). The endogenous peroxidase was blocked by 0.3% H_2_O_2_ for 10 min. After blocking with 5% BSA, the sections were incubated with primary antibody overnight at 4°C and incubated with horseradish peroxidase-conjugated secondary antibody for 1 h. The sections were subsequently reacted with DAB (Vector Laboratories), counterstained with hematoxylin, dehydrated and mounted. The images were collected using microscopy (BX51, Olympus, Japan). The histochemistry score (H-score) of protein expression was calculated by multiplying the staining intensity (0, negative; 1, weak; 2, moderate; and 3, strong) with the percentage of positive cells (0, 0%-10%; 1, 11%-25%; 2, 26%-50%; 3, 51%-75%; and 4, >75%). Antibodies used in IHC were as follows: AURKB (1:50, A19539, ABclonal), MAD2L2 (1:50, A4630, ABclonal), KI67 (1:50, 27309-1-AP, Proteintech), CyclinD1 (1:100, A11022, ABclonal), p53 (1:50, ab32049, Abcam).

1. **Co-immunoprecipitation (****Co-IP) assay**

Cells were washed twice with PBS and lysed on ice for 20 min in RIPA buffer containing 1% Cocktail (Servicebio). Lysed cells were cleared by centrifugation at 12000 ×g at 4  °C for 15 min. Cleared supernatants were removed, 10% was taken as the input, and the remainder was immunoprecipitated with IgG (Proteintech) or primary antibody (AURKB, MAD2L2) at 4 °C overnight on a rotator, followed by Protein A + G beads (Beyotime, Shanghai, China) for another 2h of incubation. The supernatant was taken from the beads after centrifugation. The beads were washed three times using the inhibitor-containing lysate and then boiled with Sample Loading Buffer (Beyotime) at 95 °C for 10 min. The input and the IgG and antibody eluates were separated by SDS-PAGE and immunoblotted as described above.

1. **CCK-8 assay**

Cell viability was analyzed using the Cell Counting Kit-8 (CCK-8; Servicebio) in accordance with the CCK8 assay protocols. Cells were seeded into 96-well plates at a density of 2 × 10^3^ cells/well. The time point when the cells adhered to the wall was taken as 0 h. When cultured to 0, 24, 48, 72, and 96 h, replaced the medium with fresh culture medium, added CCK-8 (10 µL/well) to the cells, and incubated at 37 °C for 1 h. We measured the absorbance of each well at 450 nm using a microplate reader (Bio-Rad Laboratories, Inc.).

1. **Cell cycle analysis**

After digestion, cells were harvested from 6-well plates, centrifuged, and rinsed with 1 mL of cold PBS. Cells were pelleted by centrifugation again. After removing the supernatant, the cell pellets were resuspended in cold 75% ethanol. After fixation overnight at 4 °C, cells were centrifuged and resuspended in 1 mL of cold PBS. After centrifugation again, the propidium iodide (10 µL PI Solution, 10 µL RNase A Solution, and 0.5 mL Staining Solution, YEASEN) was added to cells and incubated at 37 °C for 30 min in the dark. Cell cycle analysis was assessed by flow cytometry (CytoFLEX, Beckman Coulter, USA) according to standard protocols. Data were analyzed using the FlowJo 10.6.2 software.

1. **Apoptosis assay**

After washing with PBS, cells were digested using Trypsin without EDTA, and collected by centrifuge at 1000×g for 5 min. After rinsing with 1 mL of cold PBS, cells were counted. Cell suspensions containing 1~5 × 10^5^ cells were centrifuged, and rinsed with 1 mL of cold PBS. After removing the supernatant, the cell pellets were gently resuspended in 500 µL Annexin V Binding Buffer (Elabscience, Wuhan, China). Then 5 µL of Annexin V-FITC Reagent (Elabscience) and 5 µL of PI Reagent (Elabscience) were added to cells and incubated at room temperature for 20 min in the dark. After staining, the cells were examined immediately by flow cytometry. Data were analyzed using the FlowJo 10.6.2 software.

1. **Colony-forming assay**

Cells were seeded into 6-well plates at a density of 2000 cells/well. The medium was refreshed every 4 days. When cultured to 14 days, cells were washed twice with PBS and fixed in 4% paraformaldehyde for 30 min. After staining with crystal violet for 30 min, pictures were taken and the colony formation rate was determined.

1. **Senescence-associated β-galactosidase (SA-β-gal) staining**

Cells cultured in 6-well plates were washed with PBS and fixed with 1ml of β-galactosidase Staining Fixative (Beyotime) for 15 min. Fixed cells were washed 3 times with PBS and incubated in SA-β-Gal staining working solution (Beyotime) overnight at 37 °C. The 6-well plates were sealed with parafilm. Representative pictures were taken under bright-field microscopy (IX71, Olympus) and SA-β-gal^+^ cells rate was determined.

1. **Wound healing assay**

Cells were seeded into 6-well plates and were cultured to confluency. Wounds were generated by scratching cell layer with 200 µL plastic pipette tips and medium was replaced with culture medium with 5% serum. Cells were photographed at identical points at 0 h and 24 h post-scratch using a microscope (IX71, Olympus). Cell migration was assessed by measuring the gap distances between both sides of the scratch using the ImageJ software.

1. **Transwell cell invasion assay**

Cell invasion assay was performed with Matrigel-coated transwell chamber (Corning, USA) according to the standard method. The Matrigel (BD Biosciences) was thawed overnight at 4ºC. On ice, the Matrigel was diluted with serum-free culture medium at a ratio of 1:8. We added 80 µL of the diluted Matrigel into the Transwell chamber to ensure even spreading on the bottom of the chamber without the formation of air bubbles, and incubated at 37 ℃ for 3 h. The unbound Matrigel was removed. 600 µL of complete media was added to the lower chamber, and 200 µL of medium containing 1 × 10^4^ cells in serum-free RPMI-1640 was put in the upper chamber (8-mm pore size, Corning). The cells were cultured for 48 h. After taking out the chambers and removing the medium, we gently wiped the Matrigel and cells with a cotton swab. Then cells were fixed in 4% paraformaldehyde for 30 min and stained with crystal violet for 15 min. Five fields per well were randomly selected and images were acquired by a microscope (IX71, Olympus).

1. **Nude mouse xenograft assay**

Four-week-old Balb/c nude mice were purchased from the Centre of Experimental Animals at Wuhan University Medicine College (Hubei, China). All nude mice were kept in standard, infection-free housing conditions, and allowed to drink and eat freely. T24 cells were harvested in the logarithmic growth phase. After skin disinfection, each nude mouse received a subcutaneous injection of T24 cells (5 × 10^6^) resuspended in 100 µL of PBS. The cells were injected subcutaneously onto the back neck of nude mice by gently lifting the skin. The growth of tumor size (L, longest dimension; W, shortest dimension) was evaluated by the vernier caliper every five days, and tumor volumes were calculated using the formula V = L x W x W/2. After 45 days, mice were euthanized and the tumor tissues were dissected and weighed. The tumor tissues were then dissected into two parts. One part was immediately stored in liquid nitrogen for tissue protein extraction, and the other part was fixed in 4% paraformaldehyde for immunohistochemistry.

1. **Statistical analysis**

All statistical analyses and data visualization were performed using GraphPad Prism 8 software. All data were presented as the mean ± SD at least three independent experiments. The Shapiro‒Wilk test was employed to assess normality. For the data with normal distribution, Student's t-test was used for comparisons between two independent groups. And one-way analysis of variance (ANOVA) with Bonferroni's test was used to compare multiple groups of data. For the data with non-normal distribution, the Mann-Whitney U test was performed to analyze differences between two groups, and the Kruskal-Wallis H test, followed by Dunn's test was used for comparisons among multiple groups. Spearman’s correlation analysis was used to evaluate the relationship between the expressions of target genes. A p-value < 0.05 was considered statistically significant.
